# Safety and Treatment Effectiveness of a Single Autologous Protein Solution Injection in Patients with Knee Osteoarthritis

**DOI:** 10.1089/biores.2016.0014

**Published:** 2016-08-01

**Authors:** Rogier A.M. van Drumpt, Walter van der Weegen, William King, Krista Toler, Mitchell M. Macenski

**Affiliations:** ^1^ Department of Orthopedic Surgery, St. Anna Hospital, Geldrop, The Netherlands.; ^2^ Zimmer Biomet, Warsaw, Indiana.

**Keywords:** APS, IL-1, IRAP, pain, platelet-rich plasma, TNFα

## Abstract

Osteoarthritis (OA) is a common degenerative condition characterized by pain and loss of function. A pathological biochemical environment with excess inflammatory and catabolic proteins is a major contributor to OA. nSTRIDE^®^ Autologous Protein Solution (APS) is a new therapy under development for the treatment of OA. This therapy is formed from a patient's blood and contains high concentrations of anti-inflammatory and anabolic proteins. This study assessed the safety and treatment effects of APS. Eleven subjects with early to moderate OA were injected with APS. Subjects were closely monitored for adverse events (AE) following the injection. Treatment outcome measures were obtained before injection. AE and clinical outcomes were assessed at 1 and 2 weeks postinjection and 1, 3, and 6 months postinjection. There were no serious AE or AE that were reported by the investigator as greater than mild in severity. There were no AE that were related to the device. There were minor AE related to the injection procedure, including injection site discomfort (1/11), injection site joint pain (1/11), and procedural nausea (1/11), which resolved quickly and did not require treatment. Mean Western Ontario and McMaster Universities Arthritis Index (WOMAC) composite scores and pain, stiffness, and function subscale scores all showed significant improvement compared to baseline by 2 weeks postinjection. The data presented here suggest that the treatment is safe and show a complication profile that is mild and consistent with similar treatments. A single injection of APS for treatment of early to moderate knee OA led to symptom improvement over the study course. Based on these results, an adequately powered, well-controlled, randomized multicenter study to establish clinical efficacy is warranted.

## Introduction

Knee osteoarthritis (OA) is a common degenerative disease characterized by chronic pain, joint stiffness, reduced function, cartilage degradation, loss of subchondral bone, and synovial inflammation.^1–3^ Although symptoms may be alleviated with conservative therapies such as analgesic drugs, lifestyle modifications, and physical therapy, no disease modifying treatment is currently available. In the end phase of OA, joint replacement surgery is currently considered the only solution to relieve symptoms.^4^ New approaches that harness our understanding of early OA may allow for earlier intervention than joint replacement.

One mechanism of knee OA progression is a degenerative feed-forward cycle caused by pathological increases in inflammatory cytokines and catabolic factors within and adjacent to the synovial space. Inflammatory and catabolic proteins such as interleukin-1 beta (IL-1β), tumor necrosis factor alpha (TNFα), and matrix metalloproteinases (MMPs), for example, have been implicated in cartilage degradation and continued OA progression.^5^ Approaches to block these deleterious proteins could improve patients' symptoms, and perhaps the progression of the disease may be halted or even reversed. Autologous blood contains a host of proteins, which can block the action or production of inflammatory and catabolic proteins. Anti-inflammatory cytokines in blood include interleukin-1 receptor antagonist (IL-1ra), soluble interleukin-1 receptor type I (sIL-RI), soluble tumor necrosis factor receptor-type I (sTNF-RI), and soluble tumor necrosis factor receptor-type II (sTNF-RII).^6^ Likewise, anabolic growth factors from platelets and blood plasma, such as transforming growth factor beta (TGF-β) and insulin-like growth factor-1 (IGF-1), are also present in systemic blood.^7^ A strategy to overcome the pathologically high levels of proinflammatory and catabolic proteins, which contribute to OA, would be to introduce highly concentrated amounts of anti-inflammatory and anabolic proteins into the environment.

The nSTRIDE^®^ Autologous Protein Solution (APS) kit has been developed to process autologous blood to produce an output (herein referred to as APS) with high concentrations of anti-inflammatory cytokines and anabolic growth factors. APS has been shown to block the effects of inflammation in chondrocytes,^6^ macrophages,^8^ and cartilage explants.^9^ These cell and tissue culture-based experiments were performed using blood from healthy donors. Consequently, APS was prepared from 105 patients with radiographic evidence of OA, and patient medications and comorbidities were recorded. The cytokine profile of APS prepared from OA patient blood was not different from the cytokine profile of APS prepared from healthy donors.^10^ These data demonstrated that APS inhibits inflammatory cytokine signaling in cells and tissues and APS can be prepared from a wide variety of potential patients.

APS has demonstrated utility in treating OA pain in an equine study. Bertone et al. recruited 40 horses with OA, which were randomly assigned to receive APS (*n* = 20) or saline (*n* = 20). Horses' lameness grade was blindly assessed before the injection and at 1 and 2 weeks following the injection. At both postinjection time points, the lameness scores in the horses treated with APS were significantly better than the preinjection baseline and significantly better than the saline-treated group. The saline-treated group did not show any postinjection changes. Owners (not blinded) were asked to assess their horses' condition at 3 and 12 months postinjection. The degree of lameness was judged to be significantly better following APS injection at both postinjection time points.^11^ Together, the results of *in vitro* and large animal studies of APS motivated the first in-human clinical trial described in this study.

## Materials and Methods

### Subjects

Eleven subjects participated in this study (NCT01773226). Subjects had mild to moderate unilateral knee OA. Subjects must have failed at least one conservative therapy. Diagnosis was based on the American College of Rheumatology criteria^12^ using clinical signs and symptoms and a knee X-ray obtained within 6 months of screening. Inclusion also required subjects' report of knee pain at least 15 of the previous 30 days and a Kellgren–Lawrence grade of 2 or 3 for the index knee. Subjects had to be at least 40 years old, have a body mass index ≤40 kg/m^2^, have a Western Ontario and McMaster Universities Arthritis Index (WOMAC) pain subscale score of at least 10, and be willing to abstain from pain medications, except for acetaminophen, for the duration of the study.

Subjects were excluded from the study if they had other forms of arthritis or other inflammatory diseases, ipsilateral hip OA, traumatic knee injury, or surgical hardware in the index knee. If the subject experienced any arthroscopic procedure (12 months), intra-articular (IA) steroid injection (3 months), IA hyaluronic acid injection (6 months), or any other IA therapy (3 months) before screening, they were not eligible until they were outside the waiting window. Any condition that may have compromised the subjects' safety or ability to accurately assess the effects of the injection were excluded, including malignancy, diabetes, psychiatric illness, pregnant or nursing mothers, or other pathology at the injection site. This study was approved by the local ethics committee. All subjects participated in an informed consent process and indicated their understanding of the study requirements, risks, and obligations, and their willingness to participate in writing on an approved informed consent form.

### Study design

This study was an open label, prospective trial to characterize safety and assess treatment effectiveness. Informed consent was obtained from subjects likely to meet the inclusion and exclusion criteria. They were asked to abstain from analgesics 48 h before the next screening visit. At the screening visit, the subject's eligibility was established, including study-specific testing such as the WOMAC pain subscale. Subject's data, including demographics, medical history, and medication use, were also obtained during the screening visit. A treatment visit was scheduled within 8 weeks of screening.

Subject eligibility was confirmed before treatment. Subject-reported outcomes (WOMAC, Global Severity Scale), physician assessment of severity, and a knee examination were completed before treatment. Acetaminophen use during the trial was recorded. Following treatment, multiple injection-site reaction assessments were completed over a 2-h period, while the subject remained in the clinic, and all adverse events (AE) were recorded. The day after and 2 days after the injection, subjects were contacted to determine if any new AE had occurred. Clinical outcomes and AE were assessed at 1 and 2 weeks postinjection and 1, 3, and 6 months postinjection. Assessments included the WOMAC and both the subject's and physician's assessment of global change in the index knee. AE and medication use were also recorded.

**FIG. 1.**
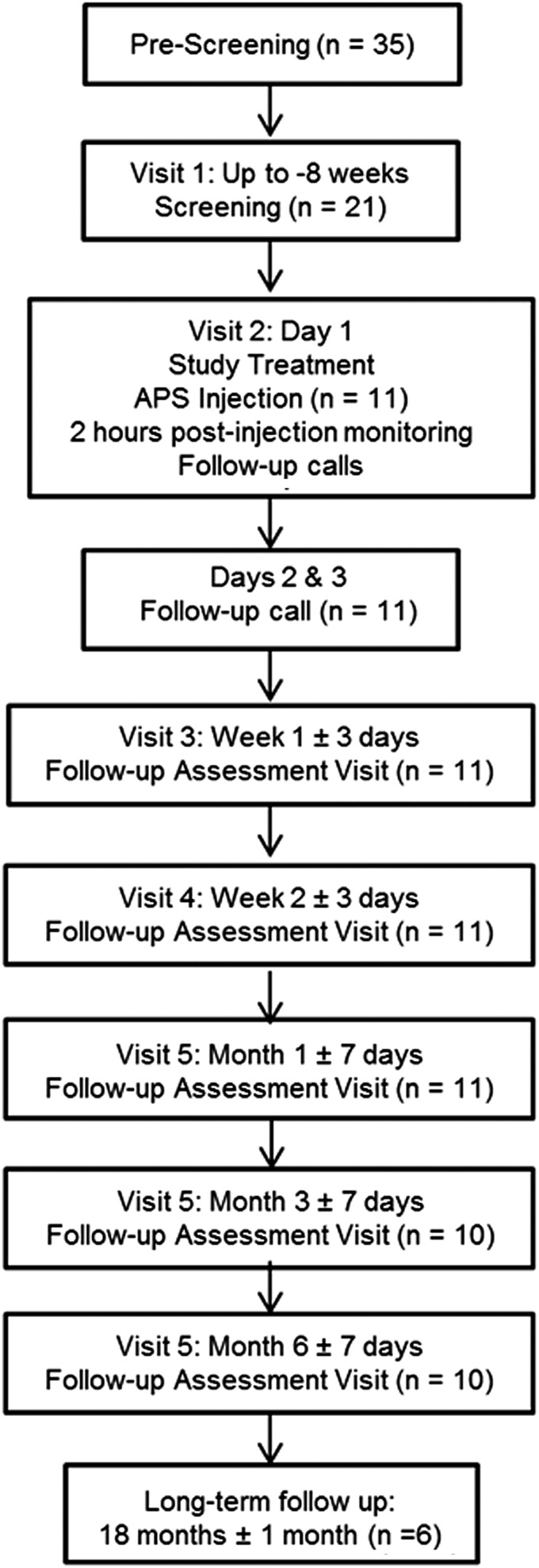
Study timeline.

Long-term analysis was performed an average of 78 weeks (18 months) after subjects were enrolled. Subjects were mailed WOMAC and Patient Global Impression-Change (PGI-C) questionnaires to be completed and returned to the study center. Two attempts were made to reach them by phone if subjects did not return their questionnaires. Long-term follow-up assessments did not include clinical visits. For subjects who returned questionnaires, the total scores and improvement compared to baseline were evaluated. In addition, OMERACT-OARSI high pain responder status was also calculated at the time of long-term follow-up (Fig. 1).

### Clinical outcome assessment

WOMAC Likert version 3.1 was used to assess knee pain, stiffness, and function. This validated scale consists of 24 items covering 3 subscales, including pain (5 items), stiffness (2 items), and function (17 items). Each item is scored from 0 (best) to 4 (worst); the worst possible score for the pain subscale is 20, the stiffness subscale is 8, and the function subscale is 68. Mean scores for all scales and the composite can range from 0 (best) to 4 (worst).

The global impression of OA severity was completed by the subject and the clinician and consists of a single seven-point assessment ranking OA severity from “Normal” to “Among the most extreme knee OA condition.” The global impression of change was also completed by the subjects and the clinician and consisted of a seven-point assessment ranking the change in OA condition from “Very much improved” to “Very much worse.” In addition, the clinician was asked to complete a two-dimensional grid ranking therapeutic effect on one axis (minimal, moderate, marked, unchanged, or worse) and side effects on the second axis (none, none of significance, significant, or outweighs therapeutic effect). The most favorable outcome would be marked therapeutic effect and no side effects, while the least desirable outcome would be unchanged or worse therapeutic effect and side effects that outweigh the therapeutic effect.

The nSTRIDE APS kit (Biomet Biologics, Warsaw, IN) contains two blood processing devices and a 30 mL vial of Anticoagulant Citrate Dextrose Solution-Formula A (ACD-A). The first of the two devices is the nSTRIDE Cell Separator. It is a plastic tube containing a tuned-density buoy, which separates cellular and platelet components of whole blood to form a cell solution. This output is further processed by the second device, the nSTRIDE Concentrator. This device is a plastic tube containing polyacrylamide absorbent beads to concentrate the cell solution and produce an injectable output, the APS. The nSTRIDE APS kit is a self-contained, sterile-packaged, single-use device.

Two APS kits were processed per subject. One volume of APS was produced to treat the index knee. The output of the second APS kit was sent to the laboratory for analysis and the results of that analysis reported in a separate publication.^13^ Blood (55 mL) was drawn into a 60 mL syringe attached to an 18-gauge butterfly apheresis needle primed with 5 mL of ACD-A. This yielded 60 mL of anticoagulated blood. The 60 mL of anticoagulated whole blood was injected into the Cell Separator and centrifuged for 15 min at 3200 RPM in a centrifuge (Biomet Biologics). When complete, the plasma was removed from the Cell Separator, using a 30 mL syringe, and was discarded. A 10 mL syringe was used to extract 2 mL of the cell solution from the Cell Separator, and the remaining cells were suspended by shaking the Cell Separator and attached syringe for 30 sec. Remaining cell solution was extracted into the syringe and then injected into the upper chamber of the Concentrator. The cell solution and beads were mixed using the attached paddle. The Concentrator was centrifuged for 2 min at 2000 RPM. The final APS volume was extracted into a 10 mL syringe and capped until injection.

The entire volume of APS (∼2.5 mL) was administered as a single IA injection. The injection area was cleaned with an antiseptic solution. A needle was positioned into the IA space under ultrasound guidance and used to aspirate and discard all available joint fluid. The syringe containing APS was attached to the needle, and the APS was injected into the synovial space of the joint. There was no specific rehabilitation protocol. Before patients left the clinic (after 2 h postinjection), they were told to minimize physical activity for 14 days postinjection (to not exceed the preinjection level of activity).

### Data analysis

Mean values were calculated with all available data. In the few cases where a data value was missing, no imputation was undertaken. Changes from baseline in the WOMAC composite score and subscales were assessed by comparing values obtained on the day of treatment to all post-treatment follow-ups. OMERACT-OARSI high pain responder status was determined if subjects had improvement in pain ≥50% and an absolute score reduction of four points or greater.^14^ These comparisons were made using two-tailed, paired *t*-tests, and significance was assumed when *p* < 0.05. AE were coded to a standard set of descriptors using the Medical Dictionary for Regulatory Activities (MedDRA).

## Results

Subjects consisted of seven males (64%) and four females (36%). Mean age of the subjects was 57.5 years (±9.5 SD) (Table 1). Six of the index knees were left knees and five were right knees. Seven (64%) of the subjects had a Kellgren–Lawrence grade of 2, while 4 (36%) had a grade of 3. Ten of the 11 subjects who started the study completed per protocol with 1 subject withdrawing after the 1-month assessment due to persistent OA symptoms.

**Table 1.** **Subject Demographics**

	Age (years)	Weight (kg)	Height (cm)	BMI (kg/m^2^)
Mean	57.5	83.6	177.1	26.6
Standard deviation	9.5	10.7	6.7	3.1
Minimum	44.6	68.0	168.0	21.0
Maximum	75.4	100.0	187.0	32.3
*N*	11	10	10	10

BMI, body mass index.

As this was a safety study, all AE were recorded even if they were not related to the device or procedure. There were no serious AE or AE reported as greater than mild in severity. There were no AE that were reported by the investigator as related to the device. There were minor AE related to the injection procedure, including injection site discomfort (1/11), injection site joint pain (1/11), and procedural nausea (1/11), which resolved quickly and did not require treatment (Table 2).

**Table 2.** **Summary of Adverse Events**

System	Event	Subjects experiencing AE^a^	AE frequency
Musculoskeletal		**6**	**16**
	Joint effusion	6	7
	Arthralgia	3	4
	Joint stiffness	3	3
	Joint instability	2	2
General disorders		**3**	**4**
	Fatigue	1	1
	Injection site discomfort	1	1
	Injection site joint pain	1	1
	Malaise	1	1
Gastrointestinal disorders		**1**	**1**
	Toothache	1	1
Procedural complications		**1**	**1**
	Nausea	1	1
Total		**9**	**22**

Bold indicates the total number of adverse events in each subsection of the table.

^a^ The value for the system header is less than the sum of the event values in that system because subjects may have experienced more than one AE.

AE, adverse events.

Global assessment of OA severity was similar between physician and subjects, with a tendency for subjects to rate initial severity more harshly than the clinicians. The physicians most frequently ranked initial severity as “Moderate” (*n* = 6), while subjects most frequently ranked initial severity as “Marked” (*n* = 7) (Table 3). Both the physician and subjects rated substantial improvement on OA severity over the course of the study. At the 3-month follow-up ratings for both the physician and the subjects, 80% of the subjects were either “Very Much Improved” or “Much Improved.” This figure would be 73% if the withdrawn subject was assumed not to have been in either of these categories. These values were also observed for the physician and the subjects at the 6-month follow-up.

**Table 3.** **Global Severity Rating for Physician and Subject at Baseline**

Rating^a^	Physician (%)	Subject (%)
Mild	1 (9.1)	0 (0.0)
Moderate	6 (54.5)	1 (9.1)
Marked	4 (36.4)	7 (63.6)
Severe	0 (0.0)	3 (27.3)

^a^ There were no instances of a rating of “Normal,” “Borderline,” or “Most Extreme Condition.”

**FIG. 2.**
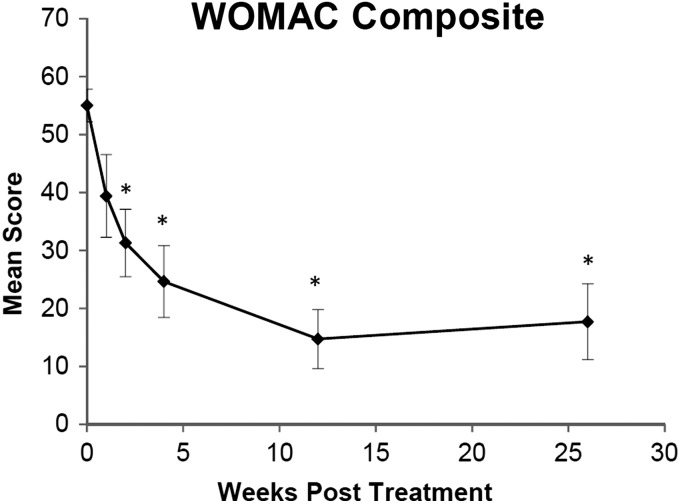
Mean WOMAC composite score as a function of time post-treatment. Error bars indicate one standard error of the mean, and asterisks indicate a significant difference (*p* < 0.05) from the pretreatment baseline (time 0). WOMAC, Western Ontario and McMaster Universities Arthritis Index.

The mean WOMAC composite score decreased by the first week post-treatment and significantly decreased by 2 weeks post-treatment (*p* < 0.01). The mean WOMAC score continued to improve through 3 months postinjection, after which it remained stable through 6 months (Fig. 2). At the 3- and 6-month follow-up, the WOMAC composite score was decreased by 70% on average. Due to missed collection and one subject withdrawing after 4 weeks, the number of subjects at each time point for WOMAC data was *t* = 0, 2 week, and 1 month: *n* = 11 and *t* = 1 week, 3, and 6 months: *n* = 10, except for WOMAC stiffness at *t* = 1 week: *n* = 9.

**FIG. 3.**
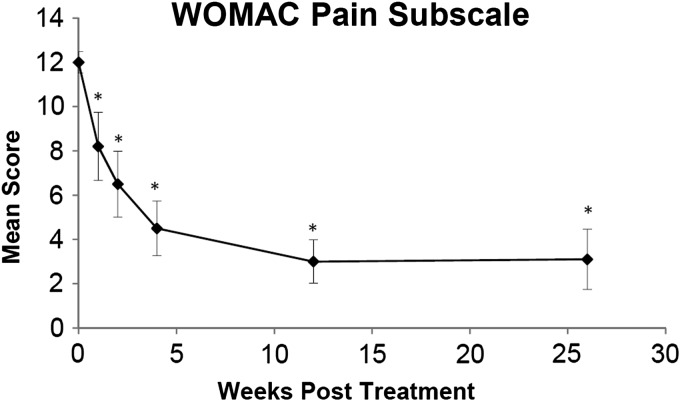
Mean WOMAC Pain Subscale score as a function of time post-treatment. Error bars indicate one standard error of the measure, and asterisks indicate a significant difference (*p* < 0.05) from the pretreatment baseline (time 0).

Mean WOMAC pain subscale scores were reduced from 12.0 before treatment to 8.2 at 1 week post-treatment (*p* = 0.05). Significant reductions in mean pain scores continued through the 3 month post-treatment visit where the mean score was reduced by 75% to 3.0. This significant reduction was maintained at 6 months post-treatment where the score was 3.1, representing a 74.2% reduction from the baseline pain score (Fig. 3).

The scores for both the WOMAC stiffness and function subscales mirrored the results of the composite scores. Mean scores for both subscales were decreased by the first week post-treatment. For the stiffness subscale, this reduction was significant (*p* = 0.03). At 2 weeks and at later time points in the study, all scores for both subscales were significantly reduced compared to baseline. As with the composite, reductions in all WOMAC subscales continued through 3 months following treatment and were maintained at the same level through 6 months postinjection (Table 4).

**Table 4.** **Western Ontario and McMaster Universities Arthritis Index Subscale Scores for Stiffness and Function**

Weeks post-treatment	Score (SEM)	Percent change	*p-*value vs. baseline
WOMAC stiffness subscale			
0	4.9 (0.3)		
1	3.8 (0.7)	22.4	0.03
2	3.1 (0.5)	36.7	<0.01
4	2.5 (0.5)	49.0	<0.01
12	1.7 (0.6)	65.3	<0.01
26	2.3 (0.6)	53.1	<0.01
WOMAC function subscale			
0	38.1 (2.3)		
1	27.8 (5.1)	27.0	0.09
2	21.9 (4.0)	42.5	<0.01
4	17.5 (4.6)	54.1	<0.01
12	10.0 (3.6)	73.8	<0.01
26	12.3 (4.7)	67.7	<0.01

WOMAC, Western Ontario and McMaster Universities Arthritis Index.

**FIG. 4.**
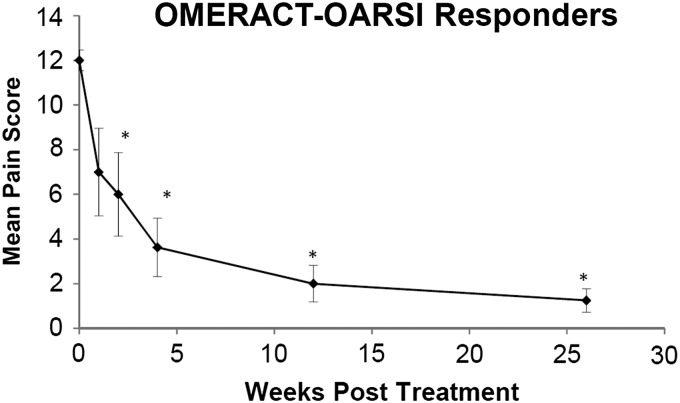
Mean WOMAC Pain Subscale score as a function of time post-treatment for subjects (*n* = 8) who were classified as OMERACT-OARSI High Pain Responders. Error bars indicate one standard error of the mean and asterisks indicate a significant difference (*p* ≤ 0.05) from the pretreatment baseline (time 0).

OMERACT-OARSI *high improvement in pain* responders were calculated as described in the Materials and Methods section.^15^ The subject who was withdrawn was classified as a nonresponder for this analysis. One week after treatment, 2 subjects met the responder criteria, and by 3 months post-treatment, 8 of 11 subjects (72.7%) were classified as high improvement in pain responders (Table 5). OMERACT-OARSI responders mean WOMAC pain subscale scores are presented in Figure 4. Responders' scores were reduced by 40% at the 1 week follow-up and were significantly lower than baseline at all other post-treatment assessments. By 3 and 6 months post-treatment, pain scores were reduced by 83% and 90%, respectively.

**Table 5.** **Number and Percentage of “High Pain Improvement Responders”**

Weeks post-treatment	*n*	Responders	Percent responders
1	10	2	20.0
2	11	5	45.5
4	11	7	63.6
12	11	8	72.7
26	11	8	72.7

OMERACT-OARSI responders defined by at least a 50% reduction in WOMAC Pain Subscale score and at least 4-point reduction in WOMAC Pain Subscale score.

Long-term follow-up was conducted after the initial study period. Total knee replacement procedures were conducted on 3 of the 11 patients after the study period (12, 12, and 24 months after enrollment, respectively) and therefore were not sent surveys. Six of the eight remaining subjects returned their questionnaires, while two subjects did not complete and return their questionnaires. The two subjects who did not return questionnaires indicated that they were still doing well and did not require treatment for their APS-injected knee. The mean WOMAC pain score was 11.8 ± 1.5 at baseline and 4.2 ± 3.3 at the 18-month time point (*n* = 6). This corresponded to a 64.4% improvement in knee pain. The mean WOMAC stiffness and function scores also had significant improvements of 58.3% (*p* = 0.03) and 61.0% (*p* < 0.01), respectively. At the long-term follow-up, two subjects rated their knee OA condition as “Very Much Improved” and four subjects rated it as “Much Improved” compared to baseline status. Finally, five of six subjects met the OMERACT-OARSI high pain responder status 18 months post-treatment.

## Discussion

This study describes the first human clinical results of APS, which is a novel autologous therapy under investigation for the treatment of knee OA. The primary goal of this study was to analyze the safety profile of APS. A secondary goal was to observe potential treatment effects of APS in patients with early to moderate OA. The results of this study provide fundamental knowledge on how the composition of APS is safe and motivates future clinical studies.

The overall safety profile of the APS was favorable, with minor complications reported. Complications were generally well tolerated by the patient and resolved without treatment. This is consistent with the theoretical idea that, as an autologous product, APS should be highly tolerable by the patient.^16^ APS contains high concentrations of leukocytes. Some authors have suggested that leukocyte-rich solutions may not be ideal for OA treatment.^17–19^ These opinions are based on cell culture models with unmatched donors^20^ and clinically insignificant (low pg/mL concentration range) differences in concentrations of inflammatory cytokines in leukocyte-rich and -poor platelet-rich plasma (PRP).^21^ The only comparative clinical study between leukocyte-rich and -poor PRP found no differences in outcomes. That comparison study used a series of three injections of Ca^2+^-activated PRP, in which the leukocyte-rich PRP was stored frozen, which is different than the APS used in this study.^22^ The clinical outcomes in this open-label study of APS were positive and robust because of the high concentration of leukocytes in the composition. However, without a proper control group, stronger comparative statements cannot be made.

The AE reported in this study were typical of those observed after any IA injection procedure and were attributed to the injection procedure rather than to APS by the investigator. A randomized, controlled trial will confirm this determination more definitively. A single injection of APS was administered in this study. Most studies of autologous therapies have used multiple injections.^22–25^ It is currently unknown if multiple injections of autologous therapies are necessary to treat knee OA, and is likely to be dependent on the composition of each product.

Following APS injection, the WOMAC composite and pain, stiffness, and function subscales all showed robust improvements in mean scores over 6 months. Similarly, both patient and physician global assessment of change was positive. The long-term durability of APS treatment persisted for a subset of patients for at least 18 months. The durability of this response could be significant, as steroids and viscosupplementation have typically shown to be effective in relieving pain for up to 6 weeks^26^ and 6 months,^27^ respectively. Unlike steroids and viscosupplementation, APS contains high concentrations of anti-inflammatory cytokines and anabolic growth factors, which could potentially alter the course of disease progression.^10^ The short-term pain relief observed could be due to the anti-inflammatory effects of APS. The long-term pain relief could be attributed to the potential disease-modifying properties of APS by improving joint homeostasis and cartilage quality. Future clinical trials, including imaging analysis, will be required to demonstrate a disease-modifying effect of APS.

The Outcomes Measures in Rheumatology Committee and the Osteoarthritis Research Society International have developed a set of responder criteria to meaningfully and objectively define, among others, “high improvement in pain” responders for OA treatment.^14^ This study not only demonstrated robust improvements in mean outcome scores but also demonstrated good treatment effects in 73% of the subjects at 3 and 6 months postinjection.

This study is limited by the lack of a control group and the small sample size, although this was sufficient for the goal of this feasibility study. A rigorous and frequent safety monitoring protocol was applied, which strengthened the results in a clinically relevant study population. To demonstrate that APS is effective in treating OA and truly can modify the disease process, more robust study designs, including randomized clinical trials, with advanced imaging of the joint are necessary.

## Conclusion

A single dose of APS for treatment of early to moderate knee OA is well tolerated by the patient, and OA symptoms were significantly improved during the study course. Based on these results, an adequately powered, well-controlled, randomized multicenter clinical study to establish clinical effectiveness is warranted.

## Abbreviations Used

ACD-A: Anticoagulant Citrate Dextrose Solution-Formula A
AE: adverse events
APS: Autologous Protein Solution
BMI: body mass index
IA: intra-articular
IGF-1: insulin-like growth factor-1
IL-1ra: interleukin-1 receptor antagonist
IL-1β: interleukin-1 beta
MMPs: matrix metalloproteinases
OA: osteoarthritis
PGI-C: Patient Global Impression-Change
PRP: platelet-rich plasma
sIL-RI: soluble interleukin-1 receptor type I
sTNF-RI: soluble tumor necrosis factor receptor-type I
sTNF-RII: soluble tumor necrosis factor receptor-type II
TGF-β: transforming growth factor beta
TNFα: tumor necrosis factor alpha
WOMAC: Western Ontario and McMaster Universities Arthritis Index

## References

[1] Buckwalter JA, Mankin HJ Articular cartilage: degeneration and osteoarthritis, repair, regeneration, and transplantation. Instr Course Lect. 1998;47:487–504 9571450

[2] Felson DT Osteoarthritis as a disease of mechanics. Osteoarthritis Cartilage. 2013;21:10–15 23041436 10.1016/j.joca.2012.09.012 PMC3538894

[3] Thorstensson CA, Andersson ML, Jonsson H, et al. Natural course of knee osteoarthritis in middle-aged subjects with knee pain: 12-year follow-up using clinical and radiographic criteria. Ann Rheum Dis. 2009;68:1890–1893 19054828 10.1136/ard.2008.095158

[4] Evans CH, Kraus VB, Setton LA Progress in intra-articular therapy. Nat Rev Rheumatol. 2014;10:11–22 24189839 10.1038/nrrheum.2013.159 PMC4402210

[5] Orlowsky EW, Kraus VB The role of innate immunity in osteoarthritis: when our first line of defense goes on the offensive. J Rheumatol. 2015;42:363–371 25593231 10.3899/jrheum.140382 PMC4465583

[6] Woodell-May J, Matuska A, Oyster M, et al. Autologous protein solution inhibits MMP-13 production by IL-1beta and TNFalpha-stimulated human articular chondrocytes. J Orthop Res. 2011;29:1320–1326 21437966 10.1002/jor.21384

[7] Eppley BL, Woodell JE, Higgins J Platelet quantification and growth factor analysis from platelet-rich plasma: implications for wound healing. Plast Reconstr Surg. 2004;114:1502–1508 15509939 10.1097/01.prs.0000138251.07040.51

[8] O'Shaughnessey K, Panitch A, Woodell-May JE Blood-derived anti-inflammatory protein solution blocks the effect of IL-1b on human macrophages in vitro. Inflamm Res. 2011;60:929–936 21687998 10.1007/s00011-011-0353-2

[9] Matuska A, O'Shaughnessey KM, King WJ, et al. Autologous solution protects bovine cartilage explants from IL-1α and TNFα induced cartilage degradation. J Orthop Res. 2013;31:1929–1935 23966313 10.1002/jor.22464

[10] O'Shaughnessey K, Matuska A, Hoeppner J, et al. Autologous protein solution prepared from the blood of osteoarthritic patients contains an enhanced profile of anti-inflammatory cytokines and anabolic growth factors. J Orthop Res. 2014;32:1349–1355 24981198 10.1002/jor.22671 PMC4134723

[11] Bertone AL, Ishihara A, Zekas LJ, et al. Evaluation of a single intra-articular injection of autologous protein solution for treatment of osteoarthritis in horses. Am J Vet Res. 2014;75:141–151 24471750 10.2460/ajvr.75.2.141

[12] Aletaha D, Neogi T, Silman AJ, et al. 2010 Rheumatoid arthritis classification criteria: an American College of Rheumatology/European League Against Rheumatism collaborative initiative. Ann Rheum Dis. 2010;69:1580–1588 20699241 10.1136/ard.2010.138461

[13] King W, van der Weegen W, van Drumpt R, et al. White blood cell concentration correlates with increased concentrations of IL-1ra and improvement in WOMAC pain scores in an open-label safety study of autologous protein solution. J Exp Orthop. 2016;3:9 26915009 10.1186/s40634-016-0043-7 PMC4747972

[14] Pham T, van der Heijde D, Lassere M, et al. Outcome variables for osteoarthritis clinical trials: the OMERACT-OARSI set of responder criteria. J Rheumatol. 2003;30:1648–1654 12858473

[15] Pham T, van der Heijde D, Altman RD, et al. OMERACT-OARSI initiative: Osteoarthritis Research Society International set of responder criteria for osteoarthritis clinical trials revisited. Osteoarthritis Cartilage. 2004;12:389–399 15094138 10.1016/j.joca.2004.02.001

[16] Filardo G, Kon E, Di MA, et al. Platelet-rich plasma vs hyaluronic acid to treat knee degenerative pathology: study design and preliminary results of a randomized controlled trial. BMC Musculoskelet Disord. 2012;13:1–8 23176112 10.1186/1471-2474-13-229 PMC3532098

[17] Anitua E, Sanchez M, Orive G, et al. The potential impact of the preparation rich in growth factors (PRGF) in different medical fields. Biomaterials. 2007;28:4551–4560 17659771 10.1016/j.biomaterials.2007.06.037

[18] Braun H, Kim H, Chu C, et al. The effect of platelet-rich plasma formulations and blood products on human synoviocytes implications for intra-articular injury and therapy. Am J Sports Med. 2014;42:1204–1210 24634448 10.1177/0363546514525593 PMC5878923

[19] Mascarenhas R, Saltzman B, Fortier L, et al. Role of platelet-rich plasma in articular cartilage injury and disease. J Knee Surg. 2014;28:3–10 25068847 10.1055/s-0034-1384672

[20] Rios DL, Lopez C, Alvarez ME, et al. Effects over time of two platelet gel supernatants on growth factor, cytokine and hyaluronan concentrations in normal synovial membrane explants challenged with lipopolysaccharide. BMC Musculoskelet Disord. 2015;20:1–12 10.1186/s12891-015-0605-3 PMC4475292 26092588

[21] Sundman EA, Cole BJ, Fortier LA Growth factor and catabolic cytokine concentrations are influenced by the cellular composition of platelet-rich plasma. Am J Sports Med. 2011;39:2135–2140 21846925 10.1177/0363546511417792

[22] Filardo G, Kon E, Pereira Ruiz MT, et al. Platelet-rich plasma intra-articular injections for cartilage degeneration and osteoarthritis: single- versus double-spinning approach. Knee Surg Sports Traumatol Arthrosc. 2011;20:2082–2091 22203046 10.1007/s00167-011-1837-x

[23] Filardo G, Kon E, Buda R, et al. Platelet-rich plasma intra-articular knee injections for the treatment of degenerative cartilage lesions and osteoarthritis. Knee Surg Sports Traumatol Arthrosc. 2011;19:528–535 20740273 10.1007/s00167-010-1238-6

[24] Sampson S, Reed M, Silvers H, et al. Injection of platelet-rich plasma in patients with primary and secondary knee osteoarthritis: a pilot study. Am J Phys Med Rehabil. 2010;89:961–969 21403592 10.1097/PHM.0b013e3181fc7edf

[25] Spakova T, Rosocha J, Lacko M, et al. Treatment of Knee joint osteoarthritis with autologous platelet-rich plasma in comparison with hyaluronic acid. Am J Phys Med Rehabil. 2012;91:411–417 22513879 10.1097/PHM.0b013e3182aab72

[26] MacMahon PJ, Eustace SJ, Kavanagh EC Injectable corticosteroid and local anesthetic preparations: a review for radiologists. Radiology. 2009;252:647–661 19717750 10.1148/radiol.2523081929

[27] Adams ME, Lussier AJ, Peyron JG A risk-benefit assessment of injections of hyaluronan and its derivatives in the treatment of osteoarthritis of the knee. Drug Saf. 2000;23:115–130 10945374 10.2165/00002018-200023020-00003

